# Cardiovascular events and mortality in chronic kidney disease in primary care patients with previous type 2 diabetes and/or hypertension. A population-based epidemiological study (KIDNEES)

**DOI:** 10.1186/s12882-022-02966-6

**Published:** 2022-11-23

**Authors:** Oriol Cunillera-Puértolas, David Vizcaya, M. Jesús Cerain-Herrero, Neus Gil-Terrón, Silvia Cobo-Guerrero, Betlem Salvador-González

**Affiliations:** 1 Institut Universitari d’Investigació en Atenció Primària Jordi Gol (IDIAPJGol), Cornellà de Llobregat, Barcelona, Spain; 2 Costa Ponent Primary Care Cardiovascular and Kidney Disease Research Group (MACAP), L’Hospitalet de Llobregat, Barcelona, Spain; 3 Bayer Pharmaceuticals, Sant Joan Despí, Barcelona, Spain; 4 Primary Care Management Costa Ponent, Primary Care Centre Can Vidalet, Institut Català de la Salut, Esplugues de Llobregat, Barcelona, Spain; 5 Primary Care Centre El Pla, Primary Care Management Costa Ponent, Catalan Institute of Health, Sant Feliu de Llobregat, Barcelona, Spain; 6 Primary Care Centre Gavarra, Primary Care Management Costa Ponent, Catalan Institute of Health, Cornellà de Llobregat, Barcelona, Spain; 7 Research Support Unit Costa Ponent, Primary Care Management Costa Ponent, Catalan Institute of Health, Cornellà de Llobregat, Barcelona, Spain

**Keywords:** Chronic kidney disease, Mortality, Cardiovascular risk, Type 2 diabetes, Hypertension

## Abstract

**Background:**

Chronic Kidney Disease (CKD), Type 2 Diabetes (T2D) and Hypertension (HTN) are frequently associated with adverse outcomes. We aimed to estimate the impact of a prior diagnosis of T2D and/or HTN on clinical characteristics, cardiovascular events (CVE) and all-cause mortality (ACM) of patients with CKD.

**Methods:**

We conducted a retrospective cohort study based on primary care electronic health records of people without atherosclerotic cardiovascular disease, aged 18–90 years with incident CKD between January 1, 2007, and December 31, 2017. The association between CKD groups classified according to prior diagnosis of T2D and/or HTN and risk of ACM and CVE at follow-up was evaluated with Cox and Fine-Gray regression models, respectively.

**Results:**

398,477 patients were included. Median age was 74 years and 55.2% were women. Individuals were classified as CKD with HTN (51.9%), CKD with T2D (3.87%), CKD with HTN/T2D (31.4%) and CKD without HTN/T2D (12.9%). In the multivariate analysis, with the CKD without HTN/T2D group as reference, the ACM Hazard Ratio (HR) was 0.74 (95%CI 0.72–0.75) for the CKD with HTN group, 0.81 (95%CI 0.79–0.83) for CKD with HTN/T2D and 1.14 (95%CI 1.10–1.19) for the CKD with T2D group. The sub distribution HRs for CVE were 1.40 (95%CI 1.34–1.47), 1.70 (95%CI 1.61–1.80) and 1.37 (95%CI 1.26–1.48), respectively.

**Conclusion:**

In patients with CKD, the risk of ACM and CVE differed in patients with previous HTN and/or T2D. These comorbidities can help identify individuals at higher risk of adverse outcomes and improve the management of patients with CKD in primary care.

**Supplementary Information:**

The online version contains supplementary material available at 10.1186/s12882-022-02966-6.

## Introduction

Chronic kidney disease (CKD) is frequently associated with adverse outcomes, including higher risk of cardiovascular events (CVE) and death [1–3]. To predict the risk of unfavourable outcomes, CKD classification is based on estimated glomerular filtration rate (eGFR) and albuminuria categories [4]. Identifying the cause of CKD is also recommended, but biopsies are only performed in specific circumstances. However, various underlying conditions can affect CKD onset and prognosis. Type 2 Diabetes (T2D) and Hypertension (HTN) are the main causes of CKD in adults, [5] and two of the main cardiovascular risk factors. A large percentage of patients with T2D and CKD also have HTN [6], and when both conditions are present, CKD prognosis may worsen [7].

The increased risk of CVE in patients with HTN, T2D and CKD is well known [8], but the interaction between T2D and HTN in the cardiovascular prognosis of patients with CKD is not yet well defined. Understanding differences in characteristics and prognostic factors of patients with CKD with diabetes and/or HTN can improve cardiovascular risk stratification and daily management.

The objective of the present study is to elucidate the impact of an underlying diagnosis of T2D and/or HTN on differences regarding clinical characteristics, prognosis and cardiovascular risk in patients with CKD without a previous diagnosis of a CVE.

## Research design and methods

### Data source

The Information System for Research in Primary Care (SIDIAP) is a well-established, anonymised, large primary care electronic health records database from Catalonia (north-east Spain), with information from primary care centres managed by the Catalan Institute of Health (catchment population of 80% of the Catalan population). The database includes demographic data, lifestyle factors, clinical diagnoses coded using the International Classification of Diseases-10th revision (ICD-10), lab values, diagnostic procedures, specialist referrals, drug prescription and hospital discharge information. The SIDIAP database is hosted by the IDIAPJGol, a primary care research institution affiliated with the Catalan Government. The SIDIAP has been validated for use in cardiovascular research [9, 10].

### Study design and participants

We conducted a retrospective cohort study based on primary care electronic health records of people aged 18–90 years with incident CKD between January 1, 2007, and December 31, 2017, seeking medical care in primary care centres of the Catalan Institute of Health. Since these were incident CKD cases without any known history of CKD for at least one year, we accessed data from January 1, 2006.

Cohort entry was defined as incident CKD evidence during the study period, verified as: (1) Two eGFR values < 60 mL/min/1.73m^2^ present for more than 90 days; (2) two abnormal urine albumin values (albumin to creatinine ratio (ACR) values ≥ 3.4 g/mmol or albumin ≥ 20 mg/L or ≥ 30 mg/day) present for more than 90 days; or (3) ICD-10 codes indicative of CKD (Supplementary Table 1).

When using eGFR or albuminuria values as the qualifying definition for eligibility, we considered as index date the date of the second abnormal measurement.

Exclusion criteria applied to patients with any previous CKD evidence as defined above. Individuals with previous atherosclerotic cardiovascular disease (angina and myocardial infarction [ICD-10: I20 - I24], ischemic or haemorrhagic stroke [I63, I64, I67] or transient ischemic attack [G45, G46], and peripheral arterial disease [I70]) were also excluded.

Subjects were followed up until transference out of the SIDIAP, or until the end of the study period (December 31, 2018).

### Exposure, outcomes and covariates

Patients were classified at baseline (first evidence of CKD) into four mutually exclusive groups according to T2D and HTN prior diagnoses: CKD with HTN, CKD with T2D, CKD with HTN/T2D (both), and CKD without HTN/T2D (none). T2D was defined according to an algorithm using ICD-10 codes [E10, E11, E12, E14 and subcategories], treatment patterns, age at diagnosis and lab values (two fasting plasma glucose ≥ 126 mg/dL (7.0 mmol/L) or glycated haemoglobin (HbA1c) ≥ 6.5%) (Supplementary Fig. 1). HTN was based on ICD-10 codes [I10, I11and subcategories], or mean blood pressure (BP) on two consecutive measurements with systolic BP (SBP) ≥ 140 and/or diastolic BP (DBP) ≥ 90 mmHg separated at least 1 week.

The outcomes were time from CKD evidence to (1) death, and (2) CVE (myocardial infarction, unstable angina or angina, non-haemorrhagic cerebrovascular disease or transient ischemic attack) (see Supplementary Table 2 for detailed definition).

The covariate definition included baseline data on age, sex, socio-economic index quintiles [11], urban or rural area, years since diagnosis of T2D or HTN and complications, CKD severity based on eGFR and albuminuria [4], other specified kidney diseases, autoimmune disease with CKD risk, heart failure, Charlson Index score, smoking status, body mass index (BMI), SBP, DBP, total cholesterol, HbA1c, anaemia and medicines taken regularly with cardiovascular or renal effects (statins, platelet aggregation inhibitors and anticoagulants, aldosterone antagonists, angiotensin-converting enzyme inhibitors (ACEI) and angiotensin II receptor antagonists (ARBs)) (Supplementary Table 2).

### Statistical analysis

Continuous variables were summarized as median and interquartile range, and categorical variables as absolute and relative frequencies.

The association of risk factors with ACM was evaluated with the Cox proportional hazards model, with estimations of ACM hazard ratio for the CKD with HTN, CKD with T2D or CKD with HTN/T2D groups compared to the CKD without HTN/T2D group, adjusted for all described co-variables . For the CVE outcome analysis, sub distribution hazard ratios were estimated from Fine-Gray models considering death as a competitive risk. For both models, transference out of the SIDIAP or end of study period were sources of right censoring, and final multivariate models were constructed from backward stepwise processes of selection of variables (based on the Akaike information criterion).

Multiple imputation of missing values ​​of co-variables was performed with the Markov Chains Multiple Imputation approach (10 imputations, 10 iterations). The number of imputations was the minimum necessary accepting a possible variability of 7% in the estimation of Standard Errors due to imputation process [12]; computational requirements did not facilitate the increase of imputations thus reducing this possible source of variability. The interactions were included in this process. Imputed variables were explored to check for quality of the imputation process. To observe the effect of data imputation, a sensitivity analysis was performed replicating the resulting models with complete case analysis. Supplementary Table 3 shows missing data values.

All analyses were performed using R-software V3.5.1 (R Foundation for Statistical Computing, Vienna, Austria).

#### Ethical approval

was obtained from the IDIAPJGol Clinical Research Ethics Committee (19/082-P).

## Results

**Fig. 1:**
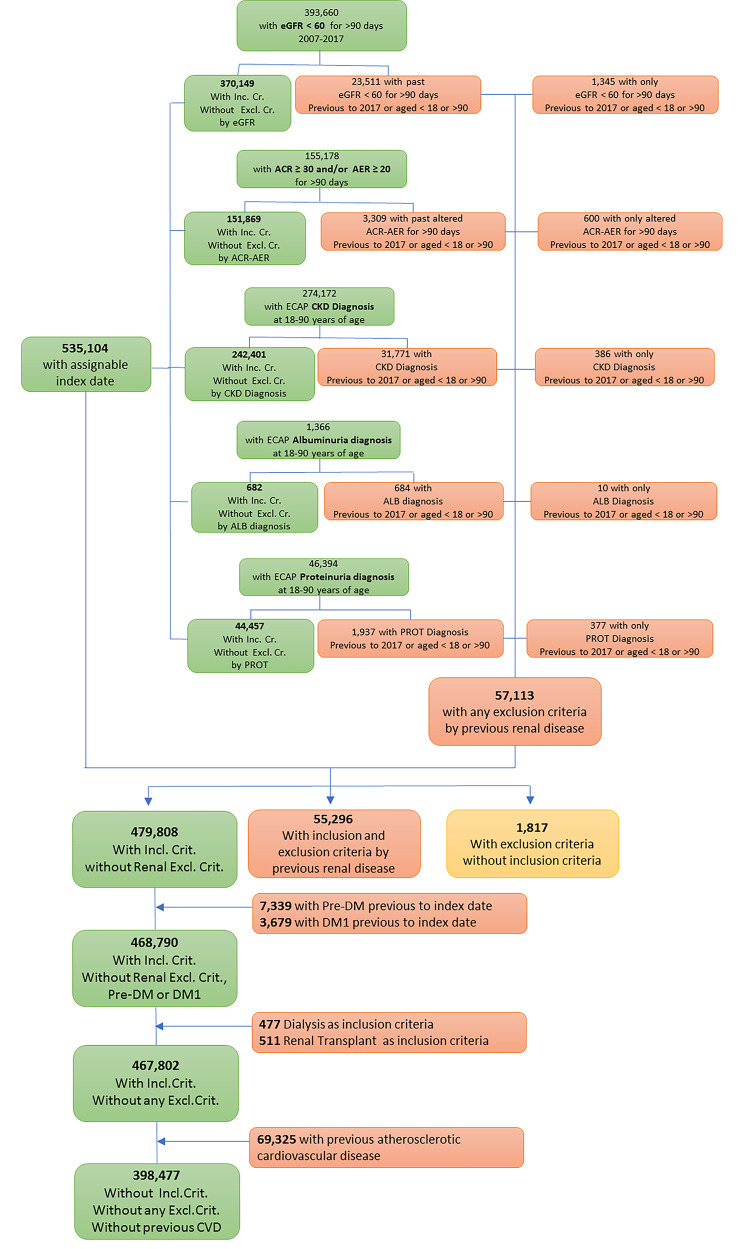
KIDNEES cohort flow-chart

A total of 398,477 individuals with incident CKD aged between 18 and 90 years were identified (Fig. 1). Median age was 74 years [IQR 65–81], and 55.2% were women. HTN was present in 83.3% and T2D in 35.3%, of whom 89.0% patients also had HTN. Individuals were distributed according to prior diagnosis of HTN and/or T2D as follows: CKD with HTN (51.9%), CKD with T2D (3.87%), CKD with HTN/T2D (31.4%), and CKD without HTN/T2D (12.9%).

Patients in the CKD with HTN group were the oldest and had the highest proportion of women (76 years [IQR 68–82] and 59.03%, respectively), as opposed to the CKD with T2D (69 years [IQR 57–78]; 38.9% women) (Table 1). The CKD with HTN/T2D and the CKD with T2D groups presented a higher proportion of lower socioeconomic quintiles and higher Charlson Index scores. Active smokers were more prevalent in the T2D-CKD group.

**Table 1:** Baseline characteristics of the KIDNEES cohort free of Atherosclerotic Cardiovascular Disease at baseline (n = 398,477) (pooled imputed data)

		CKD without HTN/T2D	CKD with HTN	CKD with T2D	CKD with HTN/T2D	p-value
Age (years)		70 [58, 80]	76 [68, 82]	69 [57, 78]	74 [65, 80]	< 0.001
Age (years, cat.)	*< 65*	39.74	20.61	42.61	25.30	< 0.001
	*(65, 80)*	37.01	47.97	38.19	51.22	
	*≥ 80*	23.24	31.42	19.20	23.48	
Sex	*Female*	56.55	59.03	38.94	50.27	< 0.001
MEDEA deprivation index	*Rural*	24.43	25.52	23.40	24.53	< 0.001
	*Least deprived quintile*	18.13	16.07	13.35	12.91	
	*Second quintile*	16.05	15.34	15.11	14.27	
	*Third quintile*	14.95	15.36	14.57	15.53	
	*Forth quintile*	13.90	14.57	15.87	16.27	
	*Most deprived quintile*	12.54	13.14	17.70	16.49	
Expanded CHARLSON INDEX SCORE	*Non comorbidity*	64.57	68.31	54.05	51.05	< 0.001
	*Low*	29.38	26.47	34.88	37.08	
	*High*	6.05	5.21	11.08	11.87	
Opht/ neur complications		0.07	0.27	10.33	13.53	< 0.001
Hypertensive heart disease		0.00	2.71	0.00	3.16	< 0.001
Other specified kidney diseases		3.43	2.28	2.32	2.09	< 0.001
Autoimmune Disease with CKD risk		0.59	0.34	0.38	0.20	< 0.001
Smoking status	*Non smoker*	66.20	72.30	53.90	65.28	< 0.001
	*Smoker*	17.10	11.37	23.47	13.54	
	*Former smoker*	16.70	16.34	22.63	21.18	
Obesity	*Underweight*	1.53	0.42	0.63	0.16	< 0.001
	*Normal*	31.64	17.78	20.22	11.85	
	*Overweight*	43.64	43.31	43.21	38.72	
	*Class 1 obesity*	17.96	27.41	25.16	31.29	
	*Class 2/3 obesity*	5.23	11.08	10.78	17.98	
Heart Failure		4.60	6.02	6.42	7.83	< 0.001
Atrial Fibrillation		6.97	9.83	8.10	10.49	< 0.001
Hypercholesterolemia		44.20	43.29	29.23	27.36	< 0.001
Systolic blood pressure	*≥ 140 mm Hg*	14.93	37.03	16.78	42.95	< 0.001
Diastolic blood pressure	*≥ 90 mm Hg*	4.83	9.81	4.06	9.74	< 0.001
Anemia		17.10	18.04	20.66	24.29	< 0.001
Statins		30.91	41.49	49.50	59.70	< 0.001
Platelet / Anticoagulant		22.64	32.40	38.54	51.57	< 0.001
Angiotensin-converting enzyme inh.		12.63	57.18	23.42	63.59	< 0.001
Angiotensin II receptor antagonists		7.09	36.01	11.84	43.37	< 0.001
Aldosterone antagonists		4.21	4.06	6.82	5.89	< 0.001

Other specified kidney diseases and autoimmune diseases with CKD risk were more frequent in the CKD without HTN/T2D group. Patients in the CKD with HTN/T2D group were more likely to present complications due to T2D and HTN, obesity, heart failure (HF) and atrial fibrillation. All these conditions were less frequent in the CKD without HTN/T2D group.

BP control was poorer in patients with HTN, with or without T2D, and cholesterol control was higher in patients with T2D. Statins, platelet aggregation inhibitors (PAI) and/or anticoagulant drugs, ACEIs and ARBs use was higher in the HTN groups, especially in the CKD with HTN/T2D group (Table 1). Aldosterone antagonists were more frequent in the CKD with T2D group.

The median time span from T2D and/or HTN diagnosis to renal disease was shorter for CKD with T2D (4.38 years; IQR 1.89–8.14) than for CKD with HTN (5.62 years; IQR 2.32–9.87) (Table 2). The CKD with HTN/T2D group was exposed to both conditions for 4.31 years [1.72–7.82] (separately, 5.90 years [2.94–9.74] to T2D and 6.77 years [3.18–11.03] to HTN). In most patients, the time between diagnosis of HTN or DM and CKD was under 10 years (73.4% and 77.1% respectively), and in 42.8% and 44.4%, respectively, less than 5 years. Percentages were higher in patients with only HTN or T2D, where 45.7% and 54.8% had CKD within 5 years (Table 2).

**Table 2:** Time span from T2D and/or HTN diagnosis to renal disease evidence and renal function parameters at baseline in the KIDNEES cohort free of Atherosclerotic Cardiovascular Disease (n = 398,477)

	CKD without HTN/T2D	CKD with HTN	CKD with T2D	CKD with HTN/T2D	p value
**Time Span from T2D and/or HTN diagnosis (years of evolution)**					
T2D	-	-	4.38 [1.89, 8.14]	5.90 [2.94, 9.74]	< 0.001
HTN	-	5.62 [2.32, 9.87]	-	6.77 [3.18, 11.03]	< 0.001
T2D and HTN	-	5.62 [2.32, 9.87]	4.38 [1.89, 8.14]	4.31 [1.72, 7.82]	< 0.001
T2D < 5 years (%)	-		54.8	43.1	
HTN < 5 years (%)		45.7		38.0	
T2D < 10 years (%)	-		83.7	76.3	
HTN < 10 years (%)	-	75.6		69.7	
**Renal function parameters (pooled imputed data)**					
eGFR severity (mL/min/1.63m^2^)					< 0.001
*< 15*	0.56	0.34	0.33	0.25	
*15–29*	2.15	2.69	2.02	2.19	
*30–44*	10.69	16.07	8.82	12.40	
*45–59*	65.69	63.01	37.63	46.47	
*60–89*	11.33	11.86	22.45	23.75	
*≥ 90*	9.58	6.03	28.76	14.94	
Albuminuria severity (%)					< 0.001
*normal to mildly increased* ^*1*^	72.98	70.04	39.11	48.09	
*moderately increased* ^*2*^	23.37	26.87	54.99	45.52	
*severely increased* ^*3*^	3.65	3.10	5.90	6.38	

^*1*^ *Albumin to creatinine ratio (ACR) < 30 mg/g or albumin < 20 mg/L;* ^*2*^ *moderately increased: ACR 30–300 mg/g or albumin 20–200 mg/L;* ^*3*^ *severely increased: ACR > 300 mg/g or albumin > 200 mg/L*

The phenotypic characteristics of CKD differed by groups (Table 2). The proportion of patients with eGFR < 60 was higher in the CKD with HTN group (82.1%) and CKD without HTN/T2D group (79.1%) than in the CKD with HTN/T2D (61.3%) and CKD with T2D (48.8%) groups. Patients with T2D were more likely to have moderately and severely increased albuminuria (60.9% in CKD with T2D, 51.9% in CKD with HTN/T2D, 30.0% in CKD with HTN and 27.0% in CKD without HTN/T2D).

Only a small percentage of patients were referred to nephrologists at baseline (within one month from CKD evidence): CKD without HTN/T2D, 1.89%; CKD with HTN, 1.17%; CKD with T2D, 0.81%; and CKD with HTN/T2D, 0.89%.

### All-cause mortality by CKD group

Over a mean follow-up of 5.82 years, 105,782 (26.6%) patients died. The overall crude mortality rate was 4,561 deaths/100,000 persons per year, highest in the CKD with T2D group (5,824/100,000), followed by CKD with HTN/T2D (5,097/100,000), CKD without HTN/T2D (4,330/100,000), and CKD with HTN (4,228/100,000) (Table 3).

**Table 3:** Crude mortality and Cardiovascular Events (CVE) rates at follow-up in the KIDNEES cohort free of Atherosclerotic Cardiovascular Disease at baseline (n = 398,477)

		Mortality		CVE	
		**CR** ^**1**^	**Mean follow-up** ^2^	**CR** ^**1**^	**Mean follow-up** ^2^
**Overall**		4,561	5.82	1,146	5.61
**Group**	CKD without HTN/T2D	4,330	5.40	653	5.29
	CKD with HTN	4,228	6.05	1,020	5.86
	CKD with T2D	5,824	5.05	1,199	4.87
	CKD with HTN/T2D	5,097	5.71	1,561	5.44

1. Crude rate: number of events per 100,000 persons per year

2. Years

**Fig. 2:**
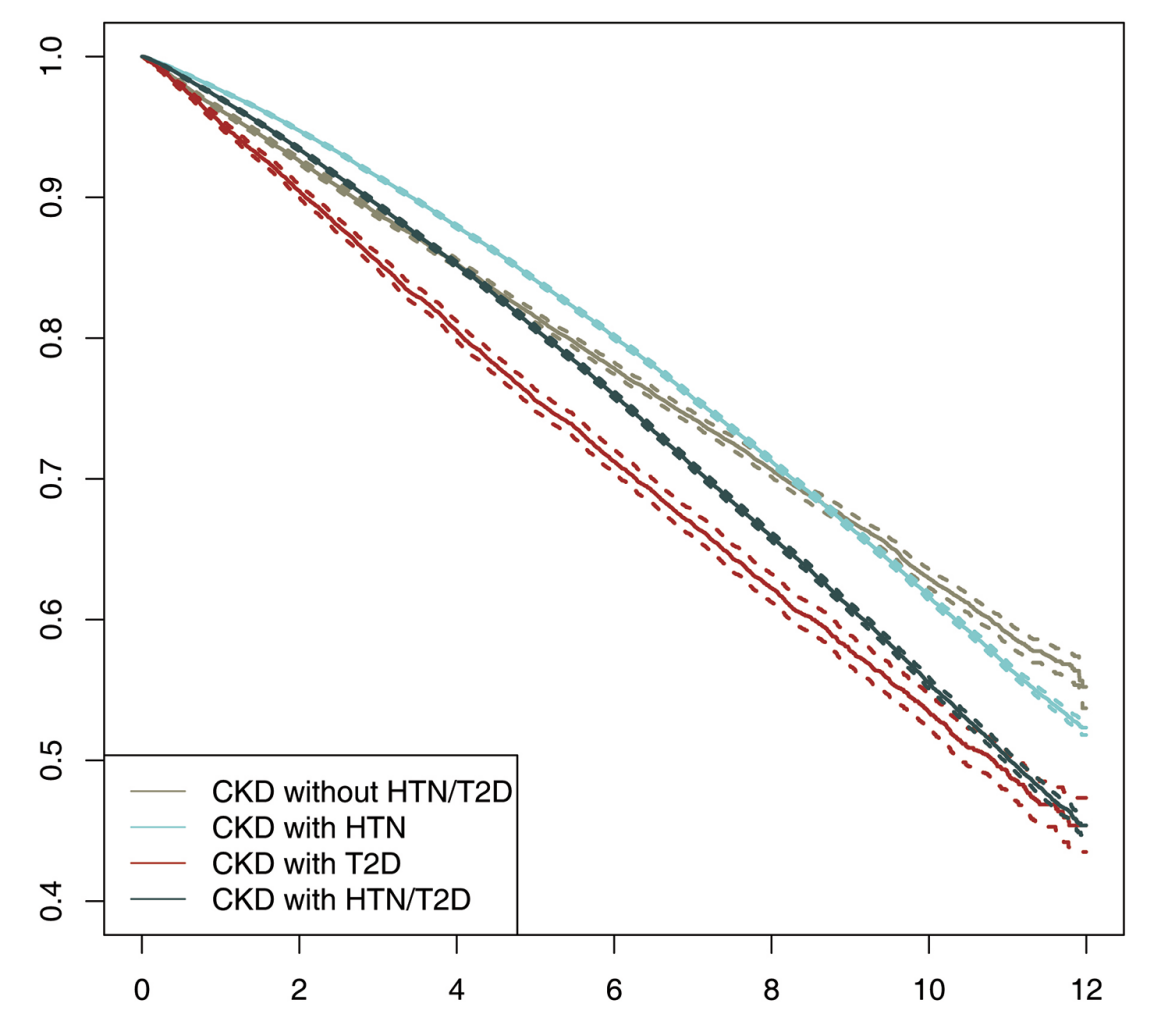
Kaplan-Meier curves on mortality by CKD-group.

**Fig. 3:**
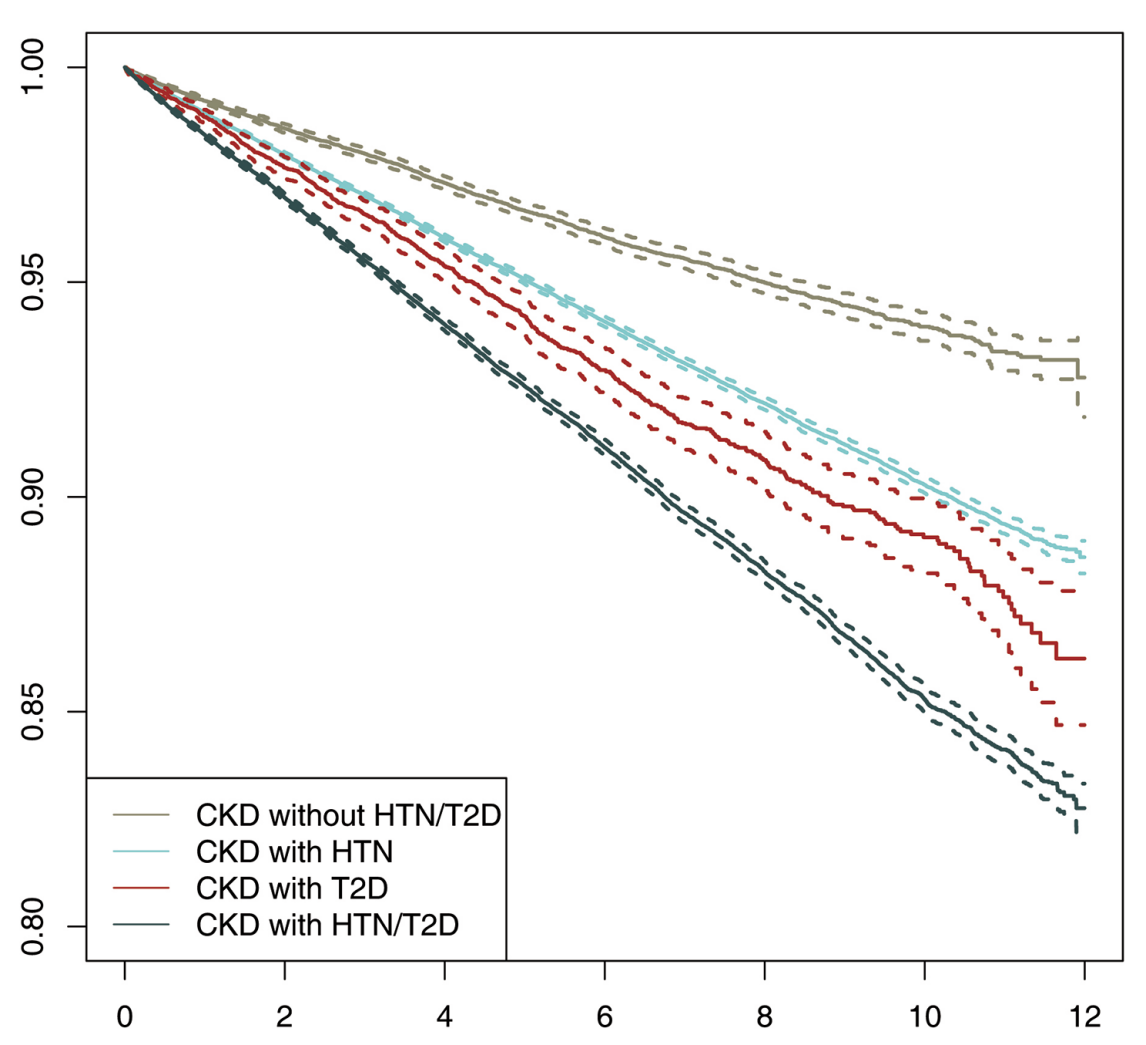
Kaplan-Meier curves on Cardiovascular Events by CKD-group.

Figure 2 shows the Kaplan-Meier mortality curves by CKD group. The higher mortality in the CKD with T2D group was distinct at the beginning, but closer to the HTN/T2D at the end of follow-up.

Supplementary Table 4 shows bivariate association of factors with ACM. In addition to the patients with T2D and those with HTN/T2D, the proportion of deaths was slightly higher in patients with complications from T2D and HTN, poorly controlled BP and HbA1c, and higher in patients with baseline eGFR < 45, anaemia, autoimmune disease with CKD risk and severe albuminuria. In contrast, mortality was lower in patients with other specified kidney diseases. Patients who died presented shorter time spans from diagnosis of T2D and/or HTN at the time of the CKD diagnosis, especially when both T2D and HTN were present (Supplementary Table 5).

In the multivariate analysis, using the CKD without HTN/T2D group as reference, the mortality risk was lower in CKD with HTN (HR 0.74; 95%CI 0.72–0.75) and CKD with HTN/T2D (HR 0.81; 95%CI 0.79–0.83), and higher for the CKD with T2D group (HR 1.14; 95%CI 1.10–1.19) (Table 4 -see Supplementary Table 6 for the full model-). Non controlled HbA1c further increased the risk of death, but not poorly controlled blood pressure. Supplementary Table 7 shows the multivariate analysis without variable selection.

**Table 4:** Multivariate adjusted hazard ratios (HR) for mortality, from a Cox proportional hazard model, associated with CKD groups, estimated glomerular filtration rate (eGFR), albuminuria categories and non-controlled HbA1c, in the KIDNEES cohort free of Atherosclerotic Cardiovascular Disease, adjusted for covariables resulting in the variable selection process (n = 398,477)

		HR	Low CI	Up. CI	p value
Group	*CKD without HTN/T2D*		(Ref.)		
	*CKD with HTN*	0.74	0.72	0.75	< 0.001
	*CKD with T2D*	1.14	1.10	1.19	< 0.001
	*CKD with HTN/T2D*	0.81	0.79	0.83	< 0.001
eGFR severity (mL/min/1.63m^2^)	*< 15*	2.39	2.19	2.61	< 0.001
	*15–29*	1.79	1.73	1.87	< 0.001
	*30–44*	1.51	1.46	1.55	< 0.001
	*45–59*	1.13	1.10	1.16	< 0.001
	*60–89*		(Ref.)		
	*≥ 90*	0.82	0.78	0.85	< 0.001
Albuminuria severity	*normal to mildly increased*		(Ref.)		
	*moderately increased*	1.40	1.37	1.44	< 0.001
	*severely increased*	1.83	1.75	1.92	< 0.001
Non controlled HbA1c		1.15	1.12	1.17	< 0.001

HR: Pooled Hazard Ratios. CI: Confidence Interval. CKD: Chronic kidney disease. HTN: Hypertension. T2D: Type 2 Diabetes Mellitus

Model adjusted by: age, sex, socioeconomic deprivation index, other primary renal disease, autoimmune disease with CKD risk, Smoking status, Obesity, Heart failure, anemia, hypercholesterolemia, statins, platelet and anticoagulants, aldosterone antagonists, angiotensin converting enzyme inhibitors, Charlson comorbidity. Estimation of covariable effects presented in Supplementary table 6. Model resulting from stepwise backwards selection process based on Akaike Information Criteria starting from model presented in supplementary table 7.

**Table 5:** Multivariate adjusted subdistributional hazard ratios (sHR) for Cardiovascular Event (CVE), considering death as a competitive risk, associated with CKD groups, estimated glomerular filtration rate (eGFR), albuminuria categories, and non-controlled blood pressure and HbA1c in the KIDNEES cohort free of Atherosclerotic Cardiovascular Disease adjusted for covariables resulting in the selection process (n = 398,477).

		sHR	Low CI	Up. CI	p value
Group	*CKD without HTN/T2*		(Ref.)		
	*CKD with HTN*	1.40	1.34	1.47	< 0.001
	*CKD with T2D*	1.37	1.26	1.48	< 0.001
	*CKD with HTN/T2D*	1.70	1.61	1.80	< 0.001
eGFR severity (mL/min/1.63m2)	*< 15*	0.93	0.73	1.19	0.561
	*15–29*	1.02	0.92	1.14	0.663
	*30–44*	1.19	1.12	1.27	< 0.001
	*45–59*	1.06	1.00	1.12	0.073
	*60–89*		(Ref.)		
	*≥ 90*	0.94	0.89	0.99	0.016
Albuminuria severity	*Normal to mildly increased*		(Ref.)		
	*Moderately increased*	1.20	1.10	1.30	0.001
	*Severely increased*	1.38	1.24	1.53	< 0.001
Systolic blood pressure	*≥ 140 mm Hg*	1.15	1.12	1.19	< 0.001
Diastolic blood pressure	*≥ 90 mm Hg*	1.10	1.04	1.16	< 0.001
Non controlled HbA1c		1.35	1.30	1.40	< 0.001

Model adjusted by: age, sex, socioeconomic deprivation index, autoimmune disease with CKD risk, Smoking habit. Obesity. Heart failure, anemia, hypercholesterolemia, platelet and anticoagulants, angiotensin converting enzyme inhibitors, angiotensin II receptor antagonists. Estimation of covariable effects presented in Supplementary table 9. Model resulting from stepwise backwards selection process based on Akaike Information Criteria starting from model presented in supplementary table 10.

Sensitivity complete-case analysis did not alter the relationship between CKD groups (Supplementary Table 11).

## Discussion

In this primary care cohort based on electronic health records of 398,477 people without atherosclerotic cardiovascular disease with incident CKD, a prior diagnosis of HTN and/or T2D was associated with the risk of mortality and CVE. After adjusting for multiple factors associated with HTN, T2D and CKD, the risk of death was higher in the group with T2D than in the group with HTN. The mortality risk for the group with both HTN and T2D was lower than for the T2D, and higher than for the HTN group. For CVE, the risk was similar in CKD with HTN or T2D, and increased when both were present. Notably, the risk associated with previous diagnosis was independent of other factors, as blood pressure, glycaemic control, or CKD phenotype. Moreover, timespan to CKD was shorter in people at increased risk of adverse outcomes. HTN and T2D, the main causes of CKD in adults [5], are frequent underlying conditions in people with CKD and were present in 83.3% and 35.3% of cases respectively in the present cohort. The prevalence of HTN in T2D patients was even higher (89.0%), and therefore the percentage of patients with only T2D was low (3.87%). These values compare to the results of similar primary care cohorts, which have described prevalence of HTN in patients with T2D and CKD of 85.4% [13] and 88.6% [14].

The characteristics of individuals differed according to CKD groups. Patients with only T2D were younger, with higher prevalence of males and smokers, and a lower prevalence of obesity compared with the CKD with HTN/T2D group. Both groups presented a high prevalence of comorbidities, HF and anaemia. In contrast, individuals in the CKD with HTN group were older, more frequently female and had lower prevalence of smokers and comorbidities.

There were also differences in the CKD phenotype. Albuminuria was more common in people with a prior diagnosis of T2D, especially those without HTN, whereas low eGFR was more commonly associated with isolated HTN or CKD without HTN/T2D. These might reflect variation in pathophysiology. In most patients with essential hypertension, biopsies show benign nephrosclerosis, characterized by a slow, progressive thickening and sclerosis of the renal resistance vessels [15]. In T2D, renal lesions are more heterogeneous and many patients have mild or absent typical glomerular lesions. Interestingly, a high number of non-diabetic renal diseases isolated or superimposed on classic diabetic nephropathy lesions have been reported, possibly reflecting the effect of additional factors such as hypertension and aging [16].

The higher mortality risk associated with CKD is well known [1, 3]. In a previous study, we performed comparisons of mortality risk in patients with CKD with/without T2D [14]. Moderate CKD (eGFR 30–50 mL/min) and T2D similarly increased the risk of death (HR 1.40 [95%CI 1.25–1.56] in CKD, 1.49 [95%CI 1.37–1.62] in T2D). The risk was higher when both CKD and T2D were present (2.19 [95%CI.91-2.51]) [14]. In the Kaiser Permanente cohort, T2D patients were 1.5-3 times more likely to die from any cause than patients without T2D in all levels of eGFR and albuminuria [17].

In people with HTN, although all-cause and cardiovascular mortality is higher than in those without, a meta-analysis showed that the associations of eGFR and ACR with mortality were stronger in patients without hypertension than in patients with hypertension [18].

Several studies have reported higher risks of death associated with HTN and T2D in individuals with CKD, either similar (HR 1.25 [95%CI 1.12–1.39] and 1.57 [95%CI 1.29–1.92], respectively) [19], or higher for T2D than for HTN (HR 1.11 [95%CI 1.02–1.21] and 1.61 [95%CI 1.52–1.70], respectively) [20].

However, the interaction between HTN and T2D, two frequently coexisting conditions in CKD, has not been fully evaluated and previous studies did not considered separately HTN and T2D diagnosis and control. The pathophysiology of HTN and CKD are intertwined. Poorly controlled blood pressure in patients with CKD is associated with progression of renal impairment.

According to our results, the risk of death associated with a prior diagnosis of T2D was higher when presented alone than when accompanied by HTN and increased further with poor glycaemic control but not blood pressure. This could suggest clinical differences in diabetes, with poorer prognosis in patients with T2D without HTN presenting earlier incident CKD at a younger age. Moreover, differences on the type of CKD, and consequently prognosis, may also exist in T2D individuals with or without HTN or in relation with the older age of the individuals in the group with HTN/T2D.

In contrast, the risk of CVE was similar for HTN and T2D, and it increased when both were present. Moreover, the risk of CVE was amplified with poor glycaemic or blood pressure control.

The known excess mortality risk in T2D has been currently described at about 15% [21, 22]. When adjusting for SBP and other risk factors, the association increased between T2D and ACM, but not for cardiovascular mortality [22].

These results in a population with mainly moderate kidney disease compare with the meta-analysis of risk factors for CVE and death in individuals with eGFR < 30, where both diabetes (HR 1.41; 95%CI 1.30–1.53) and SBP ≥ 140 mmHg (HR 1.09; 95%CI 1.04–1.15) increased the risk of CVE, but only diabetes (HR 1.12; 95%CI 1.03–1.22) increased the risk of death [23].

In another study using electronic health records in individuals with CKD with/without diabetes and no prior CVE [24], the risk of major CVE was 4.6–2.4 times higher in patients with T2D. HTN diagnosis increased by 10% the risk of CVE in patients without diabetes (HR 1.09; 95%CI 1.03–1.15), but it did not have a significant effect in patients with concomitant T2D (1.12; 0.99–1.27). Moreover, the increase in risk was higher in patients with higher BP measures and no T2D than in patients with T2D [24]. HTN is highly prevalent in T2D. In the current analysis, we differentiated between HTN diagnosis and BP control which can underscore this effect, showing no differences between CKD with HTN and CKD with T2D.

Interestingly, the time between the diagnosis of T2D and incident CKD was shorter than between HTN and CKD. Although the risk of CKD appears to increase notably with a T2D duration of 10 years and over [13], in approximately 75% patients of our cohort renal disease was found < 10 years from the HTN or T2D diagnosis, and in more than a third within just 5 years. Time span was even shorter when T2D presented without HTN. This could suggest a more aggressive clinical course or reflect a distinctive patient profile for T2D presenting in younger patients. Notably, time between T2D and HTN diagnosis in patients with CKD who died or developed a CVE was even shorter. Consequently, shorter timespans until CKD onset might identify individuals at higher risk of unfavourable outcomes. These results highlight the need to improve CKD screening and management, especially for albuminuria [25, 26]. In the current CKD cohort, albuminuria was assessed in 62% as stated in suplementary table 3 of cases, more frequently when a diagnosis of T2D was associated. The onset of CKD in the first years after T2D and/or HTN diagnosis can identify individuals at higher risk of adverse events, and screening should be emphasized. Moreover, although cardiovascular risk increases in patients with CKD, concomitant HTN and T2D further increases the risk, which should prompt a more intensive management that includes a better control of BP and HbA1c.

This study has some limitations. The design generates descriptions and associations, but does not establish causal relationships. Data were obtained from primary care electronic health records of people seeking medical care in the catchment area of Catalan Institute of Health primary care centres, and misdetection cannot be excluded. However, data for cardiovascular diseases in primary health care have proved to be of higher quality than for other diseases and suitable for epidemiological studies in our population [9].

Although models were adjusted for socioeconomic factors, cardiovascular risk factors and diseases, comorbidities and treatments, we cannot rule out unmeasured or residual confounding factors. In this sense, renal disease could act as a marker of multimorbidity. Moreover, we aimed to estimate the overall association of HTN and/or T2D exposure with the outcome, not to construct a prediction model.

The eGFR was estimated from serum creatinine measurements using the CKD-EPI formula, and creatinine-based estimating formulas have limitations, especially at higher eGFR. Data on race were not available and correction could not be applied, but Caucasian ethnicity is vastly predominant in the population under study. Although creatinine was measured in different labs, and different methods may be used, all have standardized creatinine calibration to be traceable to an isotope dilution mass spectrometry (IDMS) reference measurement procedure, which reduces variability [4]. Two different measures of albumin in urine were used. The multiple meta-analyses performed by the CKD Prognosis Consortium have also included different methods without modification of the results [1–3, 18].

CKD can remain asymptomatic with a variable time to diagnosis, and it is not uncommon to diagnose CKD at advanced stages, as reported in the literature [27]. People with T2D and/or HTN are usually managed by primary care professionals. Yearly measurements of creatinine are recommended in both conditions, but since more blood tests are performed in T2D for glucose monitoring, the probability of eGFR measurement increases. However, we do not believe that it has greatly affected the detected timespan differences, since they are also found in patients from the CKD with HTN/DM group. In contrast, the screening of albuminuria is included in all the electronic lab procedures for T2D in our region, but not for HTN, introducing a possible selection bias. It should be noted that these observed phenotypes are comparable to those reported in the literature [15, 16].

The patients who have survived both HTN and T2D to develop CKD may be incurring in immortal bias when analysing mortality risk in comparison with patients with only one of the two underlying conditions. However, as incident mainly early CKD was considered, we do not believe it is important.

The risk of stroke is higher in women and increases with age [28]. Patients in this primary care incident CKD cohort are more frequently elderly women and stroke may be overrepresented. Therefore, results might not apply to younger people with different patterns of cardiovascular disease. However, this is a typical primary care population and represents the majority of patients with CKD.

The study also has significant strengths. To our knowledge, it includes the largest incident CKD cohort published with a confirmed diagnosis using clinical criteria and with a long follow-up. Additionally, the use of real world data is representative of regular care. Finally, since in Catalonia, as in other regions like the UK, primary care acts as the gatekeeper of clinical care, we believe that a primary care database provides a real characterization of CKD in the population.

## Conclusion

The results of this study suggest that the risk of CVE and ACM in patients with CKD differ when there is a prior diagnosis of HTN and/or T2D. The identification of these underlying conditions can help identify individuals at higher risk of adverse outcomes and improve the management of patients with CKD in primary care.

## Electronic supplementary material

Below is the link to the electronic supplementary material.

Supplementary Material 1

## Data Availability

The datasets used and/or analysed during the current study are available from the corresponding author on reasonable request.

## List of abbreviations

**CKD**: Chronic kidney disease
**eGFR**: Estimated glomerular filtration rate
**CVE**: Cardiovascular event
**T2D**: Type 2 diabetes
**HTN**: Hypertension
**HR**: Hazard ratio
**sHR**: Sub distributional hazard ratio
