# Supplementary material for: Cardiovascular events and mortality in chronic kidney disease in primary care patients with previous type 2 diabetes and/or hypertension. A population-based epidemiological study (KIDNEES)
Source: BMC Nephrol. 2022 Nov 23;23:376. doi: 10.1186/s12882-022-02966-6 (PMC9805248; doi:10.1186/s12882-022-02966-6)
Supplement: Supplementary file 1 — Supplementary Material 1 [file 12882_2022_2966_MOESM1_ESM.pdf]

Cardiovascular events and mortality in Chronic Kidney Disease in primary care patients with previous Type 2 Diabetes and/or Hypertension. A population-based epidemiological study (KIDNEES)

Supplementary material

Sup Table 1. ICD-10 codes for Chronic Kidney Disease

Code	Diagnosis
D63.1	Anemia in chronic kidney disease<br>Erythropoietin resistant anemia (EPO resistant anemia)
E08.22	Diabetes mellitus due to underlying condition with diabetic chronic kidney disease
E09.22	Drug or chemical induced diabetes mellitus with diabetic chronic kidney disease
E10.22	Type 1 diabetes mellitus with diabetic chronic kidney disease
E11.22	Type 2 diabetes mellitus with diabetic chronic kidney disease
E13.22	Other specified diabetes mellitus with diabetic chronic kidney disease
I12	Hypertensive chronic kidney disease
I12.0	Hypertensive chronic kidney disease with stage 5 chronic kidney disease or end stage renal disease
I12.9	Hypertensive chronic kidney disease with stage 1 through stage 4 chronic kidney disease, or unspecified chronic kidney disease<br>Hypertensive chronic kidney disease NOS<br>Hypertensive renal disease NOS
I13	Hypertensive heart and chronic kidney disease
I13.0	Hypertensive heart and chronic kidney disease with heart failure and stage 1 through stage 4 chronic<br>kidney disease, or unspecified chronic kidney disease
I13.1	Hypertensive heart and chronic kidney disease without heart failure
I13.10	Hypertensive heart and chronic kidney disease without heart failure, with stage 1 through stage 4 chronic kidney disease, or unspecified chronic kidney disease<br>Hypertensive heart disease and hypertensive chronic kidney disease NOS
I13.11	Hypertensive heart and chronic kidney disease without heart failure, with stage 5 chronic kidney disease, or end stage renal disease
I13.2	Hypertensive heart and chronic kidney disease with heart failure and with stage 5 chronic kidney disease, or end stage renal disease
N08.3	Glomerular disorders in diabetes mellitus
N18	Chronic kidney disease
N18.1	Chronic kidney disease, stage 1
N18.2	Chronic kidney disease, stage 2 (mild)
N18.3	Chronic kidney disease, stage 3 (moderate)
N18.4	Chronic kidney disease, stage 4 (severe)
N18.5	Chronic kidney disease, stage 5
N18.9	Chronic kidney disease, unspecified<br>Chronic renal disease<br>Chronic renal failure NOS<br>Chronic renal insufficiency<br>Chronic uremia NOS<br>Diffuse sclerosing glomerulonephritis NOS

N19	Unspecified kidney failure<br>Uremia NOS
N25.0	Renal osteodystrophy<br>Azotemic osteodystrophy<br>Phosphate-losing tubular disorders<br>Renal rickets<br>Renal short stature Excludes2: metabolic
Z49.01	Encounter for fitting and adjustment of extracorporeal dialysis catheter<br>Removal or replacement of renal dialysis catheter<br>Toilet or cleansing of renal dialysis catheter
Z49.02	Encounter for fitting and adjustment of peritoneal dialysis catheter
Z94.0	Kidney transplant status
Z99.2	Dependence on renal dialysis

Sup Figure 1. Algorithm for Diabetes Mellitus reclassification based on ICD-10 codes [E10 for Type 1 diabetes mellitus, E11 for Type 2 diabetes mellitus, E12 and E14 and subcategories], treatment patterns, age at diagnosis and lab values (two fasting plasma glucose ≥ 126 mg/dL (7.0 mmol/L) or Hb A1C≥ 6.5%.

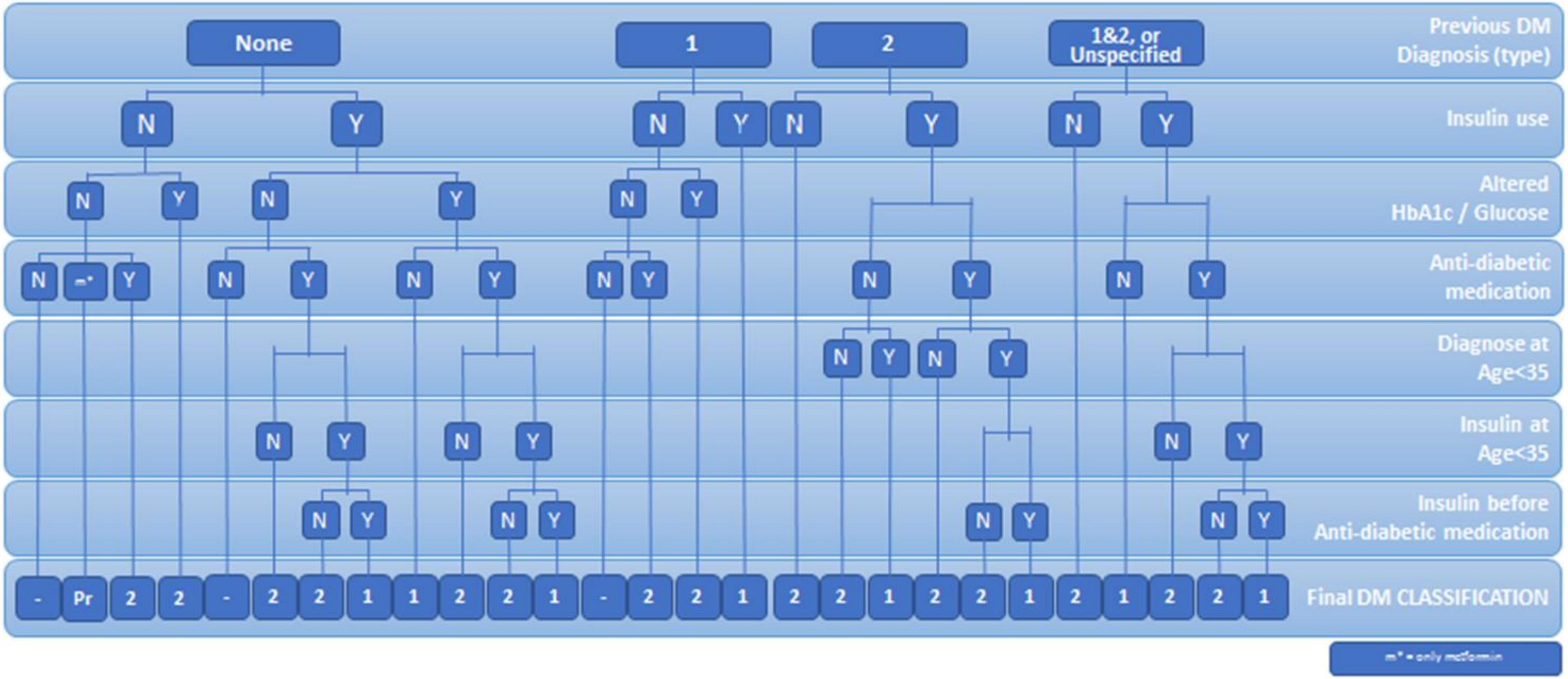

Sup Table 2. Outcomes and covariates definition.

Outcomes definition

1) All-cause mortality. Data on death obtained from administrative registers without cause specification
2) Cardiovascular events (CVE): coronary heart disease (CHD: myocardial infarction [ICD-10: I21-I24] or [ICD-9: 410, 412]), unstable angina [ICD-10: I20.0 or ICD-9: 411] or angina [ICD-10: I20]), non-haemorrhagic cerebrovascular disease [ICD-10: I63, I64 or ICD-9: 433.01, 433.11, 433.21, 433.31, 433.81, 433.91, 434.01, 434.11, 434.91] or transient ischemic attack [ICD-10: G45, G46 or ICD-9: 435]).

Covariates definition

3) SOCIODEMOGRAPHIC data: age, sex, MEDEA socio-economic index quintiles (Gac Sanit. 2008;22 (3):179-87), urban (more than 10,000 inhabitants and population density >150 inhabitants/km2), or rural (if otherwise) area.
4) T2D: years of evolution (≤ or > 10 years), and ophthalmological [E14.3, H36.0 or non-mydriatic fundus camera -AVF301- with diabetic retinopathy] and neurological complications [G63.2]
5) HTN: years of evolution (≤ or > 10 years) and presence of hypertensive heart disease [I11]
6) Expanded CHARLSON INDEX SCORE: non-comorbidity (≤ 1), low (2-3) and high (>3).
7) CARDIOVASCULAR RISK FACTORS: Smoking status (categorical: non-smoker, smoker, former smoker -ICD-10 F17 for smokers, Z72.0 for ex-smokers), obesity (according to Body Mass Index: underweight < 20.0, normal 20.0-24.9, overweight 25.0–29.9, class 1 obesity 30.0–34.9, class 2-3 obesity ≥ 35.0), hypercholesterolemia (total cholesterol ≥ 5.5 mmol/L)).
5) CARDIOVASCULAR DISEASE: heart failure [I50, I11.0, I13] , atrial fibrillation [I48].
6) PHYSICAL EXAMINATION: measurements of systolic and diastolic blood pressure (BP) (numerical, mmHg) in the last 2 years, and ankle-brachial index, weight (kg) and height (m) in the last five years
7) DISEASE WITH CKD RISK (amyloidosis [E85], systemic lupus erythematous [M32.0],
8) OTHER PRIMARY RENAL DISEASE DIAGNOSIS: acute [N00], rapidly progressive [N01], and chronic nephritic syndrome [N03], nephrotic syndrome [N04], unspecified nephritic syndrome [N05], hereditary nephropathy [N07], glomerular disorders [N08, excluded N08.3 glomerular disorders in DM], acute tubulo-interstitial nephritis [N10], chronic tubulo-interstitial nephritis [N11.9]), congenital polycystic kidney [Q61.2], IgA nephropathy [N02.8], tubulo-interstitial nephritis, not specified as acute or chronic [N12], obstructive and reflux uropathy [N13], drug- and heavy-metal-induced tubulo-interstitial and tubular conditions [N14], other renal tubulo-interstitial diseases [N15], renal tubulo-interstitial disorders in diseases classified elsewhere [N16] or AUTOIMMUNE DISEASE WITH CKD RISK (amyloidosis [E85], systemic lupus erythematous [M32.0], cryoglobulinaemia [D89.1], microscopic polyangeitis [M31.7], and Wegener disease[M31.3], multiple myeloma [C90.00].

9) LAB MEASUREMENTS. Hemoglobin (g / dL; anemia < 13 g/dL in men, <12 g/dL in women) total cholesterol (mmol / L), LDL or non-HDL (mmol / L), HDL (mmol / L), triglycerides (mmol / L), creatinine (μmol / L), glycemia (mmol / L), urate (umol / L), ionogram (mmol / L), HbA1c in diabetics (%; controlled if HbA1c <7%, or <8% in older than 80 years), albumin (mmol / L), proteins (g / L), albumin (mg/L or mg/day) or urine albumin / creatinine ratio in urine (ACR) (mg/g).
10) DRUGS OF FREQUENT USE WITH CARDIOVASCULAR OR RENAL EFFECT (dichotomic): statins [ATC code: C10AA, C10BA, C10BX], antiagregants [B01AC], anticoagulants [B01AA, B01AE, B01AF], angiotensin- converting enzyme inhibitors [C09AA, C09BA, C09BB], angiotensin II receptor antagonists [C09CA , C09DA, C09DB, C09DX], renin inhibitors [C09XA], and aldosterone antagonists [C03DA, C03DB].
11) REFERRALS: specialty, date of request, cause of referral (ICD-10 code).

Sup Table 3. Raw data missing values.

(a) Missing values by variable

Missing variable	Number of missings	%
MEDEA deprivation index	135,981	34.13
Smoking status	45,175	11.34
BMI	87,919	22.06
Blood pressure	36,804	9.24
HbA1c in diabetics	8,687	6.18
Creatinine	13,987	3.51
Albuminuria	149,704	37.57
Hemoglobin	17,756	4.46
Total cholesterol	13,597	3.41

(b) Participants with missing values by number of missing variables

Number of missing variables	Number of participants	%
0	126,554	31.76
1	108,526	27.24
2	79,979	20.07
3	36,628	9.19
4	16,538	4.15
5	13,806	3.46
6	9,738	2.44
7	2,809	0.70
8	1,123	0.28
9	2,253	0.57
10	523	0.13

Sup Table 4. Percentage of prevalence of outcomes at follow-up by baseline sociodemographic characteristics of the KIDNEES cohort free of Atherosclerotic Cardiovascular Disease at baseline (n= 398,477)

		Mortality		Cardiovascular event		
		**Exitus**	**p value**	**CVE**	**Exitus**	**p value**
Exposure group	*CKD without HTN/T2D*	23.38	<0.001	3.46	21.79	<0.001
	CKD with HTN	25.58		5.97	23.04	
	CKD with T2D	29.44		5.84	26.88	
	CKD with HTN/T2D	29.09		8.49	25.42	
Age (years)	*<65*	7.66	<0.001	4.15	6.94	<0.001
	65-79	23.56		6.97	20.88	
	≥80	49.20		7.63	44.36	
Sex	*Female*	26.51	0.581	5.90	23.79	<0.001
	Male	26.59		7.08	23.76	
MEDEA deprivation index	*Rural*	34.77	<0.001	7.64	32.99	<0.001
	Least deprived Quintile	24.15		6.52	23.46	
	Second Quintile	24.46		6.75	23.69	
	Third Quintile	24.27		7.04	23.44	
	Fourth Quintile	22.85		7.00	22.22	
	Most deprived Quintile	23.28		6.90	22.80	
Oph/ Neur complications	*No*	26.51	0.044	6.31	23.78	<0.001
	Yes	27.18		8.80	23.60	
Hypertensive heart disease	*No*	26.52	0.013	6.42	23.75	0.001
	Yes	27.66		6.82	24.88	
Other specified kidney diseases	*No*	26.77	<0.001	6.48	23.97	<0.001
	Yes	17.17		4.43	15.61	
Autoimmune Dis with CKD risk	*No*	26.51	<0.001	6.44	23.74	<0.001
	Yes	37.59		4.24	35.48	
Smoking status	*Non-smoker*	28.02	<0.001	6.96	26.82	<0.001
	Smoker	20.51		7.41	19.96	
	Former smoker	25.40		7.06	25.08	
Obesity	*Underweight*	40.61	<0.001	4.64	37.94	<0.001
	Normal	32.52		5.96	29.52	
	Overweight	26.47		6.61	23.62	
	Class 1 obesity	24.24		6.69	21.50	
	Class 2-3 obesity	22.78		6.02	20.54	
Heart failure	*No*	24.78	<0.001	6.38	22.12	<0.001
	Yes	52.31		7.25	47.95	
Atrial fibrillation	*No*	24.66	<0.001	6.26	22.07	<0.001
	Yes	44.34		8.07	39.78	
Charlson Index Score	*Noncomorbidity*	23.27	<0.001	6.55	20.58	<0.001
	Low	29.35		6.33	26.47	
	High	41.88		5.93	38.89	
eGFR severity (mL/min/1.63m^2)	*<15*	51.93	<0.001	5.14	48.99	<0.001
	15-29	55.59		6.63	51.38	
	30-44	44.28		7.81	39.80	
	45-59	25.72		6.28	23.01	
	60-89	20.41		6.69	18.02	
	≥90	8.70		5.00	7.68	
Albuminuria severity	*normal to mildly increased*	26.87	<0.001	6.51	25.95	<0.001
	moderately increased	25.46		7.72	24.50	
	severely increased	30.34		9.35	28.90	
Systolic Blood Pressure (mm Hg)	*< 140*	25.03	<0.001	5.64	22.69	<0.001
	≥ 140	29.33		7.89	25.76	
Diastolic Blood Pressure (mm Hg)	*< 90*	27.28	<0.001	6.38	24.47	<0.001
	≥ 90	19.02		6.98	16.71	
Hypercholesterolemia (mmol/L)	*<5.5*	29.14	<0.001	6.53	26.21	<0.001
	≥5.5	22.29		6.27	19.78	
HbA1c	*Controlled*	26.89	<0.001	5.97	24.23	<0.001
	Non controlled	24.53		9.13	21.13	
Anemia	*No*	22.20	<0.001	6.36	19.66	<0.001

	Yes	43.96		6.71	40.26	
Statins	*No*	28.25	<0.001	6.08	25.51	<0.001
	Yes	24.56		6.84	21.75	
Platelet inh./ Anticoagulant	*No*	20.85	<0.001	5.19	18.91	<0.001
	Yes	36.07		8.51	31.91	
Angiotensin converting enzyme inh.	*No*	25.74	<0.001	6.28	23.04	<0.001
	Yes	27.29		6.58	24.45	
Angiotensin II receptor antagonist	*No*	25.68	<0.001	5.99	23.12	<0.001
	Yes	28.26		7.31	25.07	
Aldosterone antagonists	*No*	25.37	<0.001	6.45	22.63	<0.001
	Yes	50.08		6.06	46.71	

Sup table 5. Time span from T2D and/or HTN diagnosis to renal disease evidence by outcomes at follow-up.

	Mortality			Cardiovascular event			
	**No exitus**	**Exitus**	**p value**	**No event**	**CVE**	**Exitus w/o CVE**	**p value**
T2D: Years of evolution	5.92 [2.87, 9.73]	5.26 [2.72, 9.14]	<0.001	5.94 [2.86, 9.75]	5.30 [2.72, 9.17]	5.43 [2.82, 9.23]	<0.001
HTN: Years of evolution	6.19 [2.61, 10.49]	5.65 [2.69, 9.84]	0.007	6.23 [2.63, 10.53]	5.70 [2.72, 9.89]	5.39 [2.35, 9.56]	<0.001
Group: Years of evolution	5.17 [2.01, 9.18]	4.73 [2.11, 8.56]	0.001	5.21 [2.03, 9.23]	4.77 [2.14, 8.62]	4.45 [1.83, 8.26]	<0.001

Supplementary table 6. Multivariate adjusted hazard ratios (HR) for mortality, from a Cox proportional hazard model, associated with CKD groups, estimated glomerular filtration rate (eGFR), albuminuria categories and non-controlled HbA1c, in the KIDNEES cohort free of Atherosclerotic Cardiovascular Disease, adjusted for covariables resulting in the variable selection process (n= 398,477; model presented in table 4, displaying all estimated coefficients).

		HR	Low CI	Up. CI	p value
Group	*CKD without HTN/T2D*		(Ref.)		
	CKD with HTN	0.74	0.72	0.75	<0.001
	CKD with T2D	1.14	1.10	1.19	<0.001
	CKD with HTN/T2D	0.81	0.79	0.83	<0.001
eGFR severity (mL/min/1.63m^2)	*<15*	2.39	2.19	2.61	<0.001
	15-29	1.79	1.73	1.87	<0.001
	30-44	1.51	1.46	1.55	<0.001
	45-59	1.13	1.10	1.16	<0.001
	60-89		(Ref.)		
	≥90	0.82	0.78	0.85	<0.001
Albuminuria severity	*normal to mildly increased*		(Ref.)		
	moderately increased	1.40	1.37	1.44	<0.001
	severely increased	1.83	1.75	1.92	<0.001
Non controlled HbA1c		1.15	1.12	1.17	<0.001
Age (years)	*<65*		(Ref.)		
	65-79	3.07	2.99	3.15	<0.001
	≥ 80	8.29	8.06	8.53	<0.001
Sex	*Female*		(Ref.)		
	Male	1.22	1.20	1.24	<0.001
MEDEA Deprivation index	*Rural*	1.34	1.31	1.37	<0.001
	Least deprived quintile	0.88	0.86	0.91	<0.001
	Second quintile	0.97	0.94	0.99	0.012
	Third quintile		(Ref.)		
	Forth quintile	0.97	0.95	1.00	0.031
	Most deprived quintile	1.02	1.00	1.05	0.078
Other specified kidney diseases		0.88	0.84	0.92	<0.001
Autoimmune Disease with CKD risk		1.73	1.58	1.89	<0.001
Smoking status	*Non smoker*		(Ref.)		
	Smoker	1.27	1.24	1.31	<0.001
	Former Smoker	1.16	1.14	1.19	<0.001
Obesity	*Underweight*	1.45	1.33	1.59	<0.001
	Normal		(Ref.)		
	Overweight	0.81	0.80	0.83	<0.001
	Class 1 Obesity	0.78	0.76	0.79	<0.001
	Class 2-3 obesity	0.84	0.82	0.86	<0.001
Heart failure		1.42	1.39	1.45	<0.001
Anemia		1.58	1.56	1.60	<0.001
Hypercholesterolemia		0.87	0.86	0.89	<0.001
Statins		0.87	0.86	0.88	<0.001
Platelet / Anticoagulant		1.36	1.34	1.37	<0.001
Aldosterone antagonists		1.61	1.58	1.65	<0.001
Angiotensin converting enzyme inhibitors		1.08	1.07	1.09	<0.001
Charlson	*No comorbidity*		(Ref.)		
	Low comorbidity	1.34	1.32	1.36	<0.001

High comorbidity	1.96	1.92	2.00	<0.001

Model resulting from stepwise backwards selection process based on Akaike Information Criteria starting from model presented in supplementary table 7.

Supplementary table 7. Multivariate adjusted hazard ratios (HR) for mortality, from a Cox proportional hazard model, associated with CKD groups, estimated glomerular filtration rate (eGFR), albuminuria categories and non-controlled HbA1c, in the KIDNEES cohort free of Atherosclerotic Cardiovascular Disease, adjusted for all clinically selected covariables (n= 398,477).

		**HR**	**Low CI**	**Up. CI**	**p value**
Group	*CKD without HTN/T2D*		(Ref.)		
	CKD with HTN/T2D	0.74	0.72	0.75	<0.001
	CKD with HTN/T2D	1.14	1.10	1.18	<0.001
	CKD with HTN/T2D	0.80	0.78	0.82	<0.001
eGFR severity (mL/min/1.63m^2)	*<15*	2.39	2.19	2.61	<0.001
	15-29	1.80	1.73	1.87	<0.001
	30-44	1.51	1.46	1.55	<0.001
	45-59	1.13	1.10	1.16	<0.001
	60-89		(Ref.)		
	≥90	0.82	0.78	0.85	<0.001
Albuminuria severity	*normal to mildly increased*		(Ref.)		
	moderately increased	1.40	1.37	1.44	<0.001
	severely increased	1.83	1.74	1.91	<0.001
Systolic blood pressure	*≥ 140 mm Hg*	1.00	0.99	1.02	0.556
Diastolic blood pressure	*≥ 90 mm Hg*	1.00	0.98	1.03	0.815
Non controlled HbA1c		1.15	1.12	1.17	<0.001
Age (years)	*<65*		(Ref.)		
	65-79	3.07	2.99	3.15	<0.001
	≥ 80	8.29	8.06	8.54	<0.001
Sex	*Female*		(Ref.)		
	Male	1.22	1.20	1.24	<0.001
MEDEA Deprivation index	*Rural*	1.34	1.31	1.37	<0.001
	Least deprived quintile	0.88	0.86	0.91	<0.001
	Second quintile	0.97	0.94	0.99	0.012
	Third quintile		(Ref.)		
	Forth quintile	0.97	0.95	1.00	0.030
	Most deprived quintile	1.02	1.00	1.05	0.080
Oft /neur. complications		1.02	0.99	1.05	0.216
Hypertensive heart disease		1.04	1.00	1.08	0.052
Other specified kidney diseases		0.878	0.88	0.84	0.92
Autoimmune Disease with CKD risk		1.733	1.73	1.58	1.90
Smoking status	*Non smoker*		(Ref.)		
	Smoker	1.27	1.24	1.31	<0.001
	Former Smoker	1.16	1.14	1.19	<0.001
Obesity	*Underweight*	1.45	1.33	1.59	<0.001
	Normal		(Ref.)		
	Overweight	0.81	0.80	0.83	<0.001
	Class 1 Obesity	0.77	0.76	0.79	<0.001
	Class 2-3 obesity	0.84	0.82	0.86	<0.001
Heart failure		1.42	1.39	1.45	<0.001
Anemia		1.58	1.56	1.60	<0.001
Hypercholesterolemia		0.87	0.86	0.89	<0.001
Statins		0.87	0.86	0.88	<0.001
Platelet / Anticoagulant		1.36	1.34	1.37	<0.001
Aldosterone antagonists		1.61	1.58	1.65	<0.001
Angiotensin converting enzyme inhibitors		1.08	1.07	1.10	<0.001
Angiotensin II receptor antagonists		1.00	0.99	1.02	0.528
Charlson	*No comorbidity*		(Ref.)		
	Low comorbidity	1.34	1.32	1.36	<0.001
	High comorbidity	1.96	1.92	2.00	<0.001

HR: Pooled Hazard Ratios.

Sup Table 8. Multivariate adjusted hazard ratios (HR) for mortality, from a Cox proportional hazard model, associated with CKD groups, estimated glomerular filtration rate (eGFR), albuminuria categories and non-controlled HbA1c, in the KIDNEES cohort free of Atherosclerotic Cardiovascular Disease, adjusted for covariables resulting in the variable selection process (complete case analysis; n=129.210)

		HR	Low CI	Up. CI	p value
Group	*CKD without HTN/T2D*		(Ref.)		
	CKD with HTN	0.83	0.76	0.90	<0.001
	CKD with T2D	1.15	1.04	1.27	0.005
	CKD with HTN/T2D	0.89	0.81	0.97	0.009
eGFR severity (mL/min/1.63m^2)	*<15*	2.57	2.06	3.20	<0.001
	15-29	1.78	1.64	1.94	<0.001
	30-44	1.55	1.48	1.63	<0.001
	45-59	1.16	1.12	1.21	<0.001
	60-89		(Ref.)		
	≥90	0.81	0.77	0.86	<0.001
Albuminuria severity	*normal to mildly increased*		(Ref.)		
	moderately increased	1.48	1.43	1.53	<0.001
	severely increased	2.11	1.99	2.23	<0.001
Non controlled HbA1c		1.13	1.09	1.16	<0.001
Age (years)	*< 65*		(Ref.)		
	65-79	2.91	2.76	3.06	<0.001
	≥ 80	8.22	7.78	8.69	<0.001
Sex	*Female*		(Ref.)		
	Male	1.23	1.19	1.27	<0.001
MEDEA Deprivation Index	*Rural*	1.43	1.37	1.49	<0.001
	Least deprived Quintile	0.93	0.89	0.98	0.009
	Second Quintile	0.99	0.94	1.04	0.690
	Third Quintile		(Ref.)		
	Fourth Quintile	0.97	0.93	1.02	0.238
	Most deprived Quintile	1.02	0.97	1.07	0.409
Other specified kidney diseases		0.77	0.69	0.86	<0.001
Autoimmune Disease with CKD risk		1.73	1.37	2.20	<0.001
Smoking status	*Non smoker*		(Ref.)		
	Smoker	1.31	1.25	1.38	<0.001
	Former Smoker	1.17	1.13	1.22	<0.001
Obesity	*Underweight*	2.03	1.69	2.44	<0.001
	Normal		(Ref.)		
	Overweight	0.76	0.73	0.78	<0.001
	Class 1 Obesity	0.70	0.68	0.73	<0.001
	Class 2-3 obesity	0.79	0.75	0.82	<0.001
Heart failure		1.43	1.37	1.49	<0.001
Anemia		1.54	1.50	1.59	<0.001
Hypercholesterolemia		0.94	0.91	0.97	<0.001
Statins		0.82	0.80	0.84	<0.001
Platelet inhibitors / Anticoagulants		1.33	1.29	1.36	<0.001
Aldosterone antagonists		1.72	1.64	1.81	<0.001
Angiotensin converting enzyme inhibidors		1.05	1.02	1.08	<0.00
Charlson Index Score	*No comorbidity*		(Ref.)		
	Low comorbidity	1.39	1.35	1.43	<0.001
	High comorbidity	1.91	1.83	1.99	<0.001

**Complete case version of the multiply imputed model presented in table 4*

Supplementary table 9. Multivariate adjusted subdistributional hazard ratios (sHR) for Cardiovascular Event (CVE), considering death as a competitive risk, associated with CKD groups, estimated glomerular filtration rate (eGFR), albuminuria categories, and non-controlled blood pressure and HbA1c, in the KIDNEES cohort free of Atherosclerotic Cardiovascular Disease adjusted for baseline co-variables with backwards selection process (n= 398,477; model presented in table 5, displaying all estimated coefficients).

		sHR	Low CI	Up. CI	p value
Group	*CKD without HTN/T2*		(Ref.)		
	CKD with HTN	1.40	1.34	1.47	<0.001
	CKD with T2D	1.37	1.26	1.48	<0.001
	CKD with HTN/T2D	1.70	1.61	1.80	<0.001
eGFR severity (mL/min/1.63m2)	*<15*	0.93	0.93	1.19	0.561
	15-29	1.02	0.92	1.14	0.663
	30-44	1.19	1.12	1.27	<0.001
	45-59	1.06	1.00	1.12	0.073
	60-89		(Ref.)		
	≥90	0.94	0.89	0.99	0.016
Albuminuria severity	*Normal to mildly increased*		(Ref.)		
	Moderately increased	1.20	1.10	1.30	0.001
	Severely increased	1.38	1.24	1.53	<0.001
Systolic blood pressure	*≥ 140 mm Hg*	1.15	1.12	1.19	<0.001
Diastolic blood pressure	*≥ 90 mm Hg*	1.10	1.04	1.16	<0.001
Non controlled HbA1c		1.35	1.30	1.40	<0.001
Age (years)	*<65*		(Ref.)		
	65 - 79	1.65	1.58	1.72	<0.001
	>=80	2.02	1.93	2.12	<0.001
Sex	*Female*		(Ref.)		
	Male	1.28	1.24	1.31	<0.001
MEDEA Deprivation Index	*Rural*	1.04	1.00	1.10	0.078
	Least deprived quintile	0.93	0.88	0.97	0.002
	Second quintile	0.96	0.91	1.01	0.100
	Third quintile		(Ref.)		
	Forth quintile	1.00	0.95	1.05	0.965
	Most deprived quintile	0.98	0.93	1.03	0.401
Oft /neur. Complications		1.13	1.07	1.19	<0.001
Smoking status	*Non smoker*		(Ref.)		
	Smoker	1.19	1.14	1.24	<0.001
	Former Smoker	0.97	0.93	1.00	0.085
Obesity	*Underweight*	0.91	0.69	1.21	0.523
	Normal		(Ref.)		
	Overweight	1.04	0.99	1.08	0.093
	Class 1 Obesity	1.03	0.99	1.08	0.172
	Class 2-3 obesity	0.95	0.90	1.01	0.089
Heart failure		0.96	0.92	1.01	0.121
Anemia		0.92	0.88	0.95	<0.001
Hypercholesterolemia		1.09	1.06	1.12	<0.001
Platelet inh. / anticoagulants		1.42	1.38	1.46	<0.001
Angiotensin-converting enzyme inhibitors.		0.94	0.91	0.96	<0.001
Angiotensin II receptor antagonists		1.05	1.02	1.08	0.001

Model resulting from stepwise backwards selection process based on Akaike Information Criteria starting from model presented in supplementary table 10.

Supplementary table 10. Multivariate adjusted subdistributional hazard ratios (sHR) for Cardiovascular Event (CVE), considering death as a competitive risk, associated with CKD groups, estimated glomerular filtration rate (eGFR), albuminuria categories, and non-controlled blood pressure and HbA1c, in the KIDNEES cohort free of Atherosclerotic Cardiovascular Disease, adjusted for all clinically selected covariables (n= 398,477).

		sHR	Low. CI	Up. CI	p value
Group	*CKD without HTN/T2D*		(Ref.)		
	CKD with HTN	1.39	1.31	1.48	<0.001
	CKD with T2D	1.39	1.27	1.51	<0.001
	CKD with HTN/T2D	1.71	1.60	1.84	<0.001
eGFR severity (mL/min/1.63m^2)	*<15*	0,96	0.73	1.25	0.735
	15-29	1.05	0.94	1.17	0.391
	30-44	1.20	1.12	1.29	<0.001
	45-59	1.06	0.99	1.13	0.108
	60-89		(Ref.)		
	≥90	0.93	0.87	0.98	0.010
Albuminuria severity	*normal to mildly increased*		(Ref.)		
	moderately increased	1.190	1.09	1.29	<0.001
	severely increased	1.38	1.24	1.53	<0.001
Systolic blood pressure	*≥ 140 mm Hg*	1.15	1.12	1.18	<0.001
Diastolic blood pressure	*≥ 90 mm Hg*	1.10	1.05	1.15	<0.001
Non controlled HbA1c		1.34	1.30	1.39	<0.001
Age (years)	*<65*		(Ref.)		
	65 - 79	1.65	1.59	1.71	<0.001
	≥ 80	2.02	1.94	2.10	<0.001
Sex	*Female*		(Ref.)		
	Male	1.29	1.25	1.32	<0.001
MEDEA Deprivation Index	*Rural*	1,04	1.00	1.09	0.068
	Least deprived quintile	0,93	0.88	0.98	0.007
	Second quintile	0.96	0.91	1.01	0.123
	Third quintile		(Ref.)	(ref.)	
	Forth quintile	1.001	0.96	1.05	0.964
	Most deprived quintile	0.980	0.93	1.03	0.449
Oft /neur. complications		1.14	1.09	1.20	<0.001
Hypertensive heart disease		1.058	0.98	1.15	0.173
Other specified kidney diseases		0.906	0.82	1.00	0.047
Autoimmune Disease with CKD risk		0.920	0.69	1.23	0.576
Smoking status	*Non smoker*		(Ref.)		
	Smoker	1.20	1.15	1.25	<0.001
	Former Smoker	0.98	0.94	1.02	0.274
Obesity	*Underweight*	0.92	0.70	1.20	0.537
	Normal		(Ref.)		
	Overweight	1.03	0.99	1.08	0.140
	Class 1 Obesity	1.03	0.98	1.08	0.274
	Class 2-3 obesity	0.95	0.90	1.01	0.076
Heart failure		1.04	0.99	1.09	0.148
Anemia		0.93	0.90	0.97	<0.001
Hypercholesterolemia		1.09	1.06	1.11	<0.001
Statins		1.02	0.99	1.04	0.242
Platelet inh. / anticoagulants		1,43	1.39	1.46	<0.001
Aldosterone antagonists		0.85	0.79	0.91	<0.001
Angiotensin-converting enzyme inh.		0,94	0.91	0.96	<0.001
Angiotensin II receptor antagonists		1,05	1.02	1.08	0.002
Charlson Index Score	*No comorbidity*		(Ref.)		
	Low comorbidity	0.95	0.92	0.98	<0.001
	High comorbidity	0.85	0.81	0.89	<0.001

Supplementary Table 11. Multivariate adjusted subditributional hazard ratios (sHR) for Cardiovascular Events, considering death as a competitive risk, associated with CKD groups, estimated glomerular filtration rate (eGFR), albuminuria categories and non-controlled HbA1c, in the KIDNEES cohort free of Atherosclerotic Cardiovascular Disease, adjusted for covariables resulting in the variable selection process (complete case analysis; n=129.210)

		**sHR**	**Low CI**	**Up. CI**	**p value**
Group	*CKD without HTN/T2D*		(Ref.)		
	CKD with HTN	1.37	1.22	1.55	<0.001
	CKD with T2D	1.52	1.29	1.78	<0.001
	CKD with HTN/T2D	1.73	1.53	1.96	<0.001
eGFR severity (mL/min/1.63m^2)	*<15*	0.82	0.50	1.34	0.420
	15-29	0.95	0.82	1.11	0.520
	30-44	1.13	1.05	1.22	0.002
	45-59	1.07	1.01	1.14	0.022
	60-89		(Ref.)		
	≥90	0.96	0.89	1.04	0.280
Albuminuria severity	*normal to mildly increased*		(Ref.)	(ref.)	
	moderately increased	1.23	1.17	1.30	<0.001
	severely increased	1.49	1.36	1.63	<0.001
Systolic blood pressure	*≥ 140 mm Hg*	1.19	1.14	1.24	<0.001
Diastolic blood pressure	*≥ 90 mm Hg*	1.13	1.06	1.21	<0.001
Non controlled HbA1c		1.33	1.27	1.40	<0.001
Age (years)	*<65*		(Ref.)		
	65-79	1.56	1.47	1.66	<0.001
	≥ 80	2.03	1.89	2.18	<0.001
Sex	*Female*		(Ref.)		
	Male	1.30	1.25	1.36	<0.001
MEDEA Deprivation Index	*Rural*	1.00	0.94	1.07	0.980
	Least deprived Quintile.	0.89	0.83	0.96	0.002
	Second Quintile	0.94	0.88	1.00	0.062
	Third Quintile				
	Fourth Quintile	0.97	0.91	1.04	0.430
	Most deprived Quintile	0.94	0.88	1.01	0.110
Ophthalmological / neurological complications		1.16	1.09	1.24	<0.001
Smoking status	*Non smoker*		(Ref.)		
	Smoker	1.21	1.14	1.28	<0.001
	Former Smoker	0.97	0.92	1.02	0.170
Obesity	*Underweight*	0.96	0.67	1.38	0.820
	Normal		(Ref.)		
	Overweight	1.03	0.97	1.09	0.350
	Class 1 Obesity	1.00	0.94	1.07	0.900
	Class 2-3 obesity	0.92	0.86	1.00	0.046
Heart failure		1.05	0.98	1.13	0.180
Anemia		0.94	0.89	0.99	0.013
Hypercholesterolemia		1.07	1.02	1.12	0.003
Platelet / Anticoagulant		1.36	1.30	1.43	<0.001
Angiotensin converting enzyme inhibitors		0,94	0.90	0.98	0.002
Angiotensin II receptor antagonists		1,08	1.03	1.12	<0.001

**Complete case version of the multiply imputed model presented in table 5*
